# Acute organ failure and risk of admission to intensive medical care in cancer patients: a single center prospective cohort study

**DOI:** 10.5935/0103-507X.20210085

**Published:** 2021

**Authors:** Sara Coelho, Teresa Ribeiro, Isabel Pereira, Delfim Duarte, Ana Afonso, Iolanda Meneses, Sofia Pinelas, Brigitte Pereira, Fernando Coelho, Anabela Martins, Nuno Sousa, Filomena Faria

**Affiliations:** 1 Medical Oncology Department, Instituto Português de Oncologia do Porto Francisco Gentil - Porto, Portugal.; 2 Hematological Oncology Department, Instituto Português de Oncologia do Porto Francisco Gentil - Porto, Portugal.; 3 Intensive Care Unit, Instituto Português de Oncologia do Porto Francisco Gentil - Porto, Portugal.

**Keywords:** Neoplasms, Hematologic neoplasms, Multiple organ failure, Critical illness

## Abstract

**Objective:**

To ascertain the cumulative incidence of acute organ failure and intensive care unit admission in cancer patients.

**Methods:**

This was a single-center prospective cohort study of adult cancer patients admitted for unscheduled inpatient care while on systemic cancer treatment.

**Results:**

Between August 2018 and February 2019, 10,392 patients were on systemic treatment, 358 had unscheduled inpatient care and were eligible for inclusion, and 285 were included. The mean age was 60.9 years, 50.9% were male, and 17.9% of patients had hematologic cancers. The cumulative risk of acute organ failure was 39.6% (95%CI: 35 - 44), and that of intensive care unit admission among patients with acute organ failure was 15.0% (95%CI: 12 - 18). On admission, 62.1% of patients were considered not eligible for artificial organ replacement therapy. The median follow-up time was 9.5 months. Inpatient mortality was 17.5%, with an intensive care unit mortality rate of 58.8% and a median cohort survival of 134 days (95%CI: 106 - 162). In multivariate analysis, acute organ failure was associated with 6-month postdischarge mortality (HR 1.6; 95%CI: 1.2 - 2.2).

**Conclusion:**

The risk of acute organ failure in cancer patients admitted for unscheduled inpatient care while on systemic treatment was 39.6%, and the risk of intensive care unit admission was 15.0%. Acute organ failure in cancer patients was an independent poor prognostic factor for inpatient hospital mortality and 6-month survival.

## INTRODUCTION

The incidence of cancer is estimated to be more than 3 million cases *per* year in Europe, with approximately 1.5 million cancer-related deaths.^(^1^)^ With the improvement of diagnostic tests and treatments for cancer, there has been a steady decrease in cancer-related mortality.^(^2^)^ The increasing number of patients receiving cancer treatment has resulted in more frequent adverse drug reactions, some of which are associated with acute organ failure (AOF), consequently increasing the number of cancer patients who may require admission to intensive care units (ICU).^(^3^-^5^)^

The incidence of AOF in patients on anti-neoplastic systemic treatment is unknown. It is estimated that 5% of patients with solid tumors and 15% with hematological cancer will need admission to an ICU in the early stages of their disease.^(^5^-^7^)^ Patients with advanced cancer may benefit from ICU admission.^(^8^-^11^)^ The two most common causes of ICU admission in patients with cancer are acute respiratory failure and sepsis.^(^5^)^ Although survival rates for cancer patients admitted for ICU care are lower than those of patients without comorbidities, their mortality rates are similar to those of patients with other comorbidities, namely, chronic heart failure.^(^12^)^ Early identification of organ failure and timely admission to the ICU are critical determinants of the short-term prognosis of these patients.^(^5^)^ However, the long-term outcome depends on the characteristics of the malignant disease and its prognosis, not on the severity of the acute event.^(^7^,^13^)^

The IPOPSCI-2017/01 study was designed to estimate the incidence of AOF in cancer patients on systemic anti-neoplastic treatment and to estimate the incidence of ICU admission and prognosis of these patients in the setting of the largest Portuguese comprehensive cancer center.

## METHODS

This was a prospective cohort study with consecutively sampled cancer patients admitted for in-hospital care due to a medical complication of cancer treatment at *Instituto Português de Oncologia do Porto Francisco Gentil* from August 2018 to February 2019.

The key inclusion criteria were as follows: patient age of 18 years or older; a histological or cytological diagnosis of a malignancy; active antineoplastic treatment, which was defined as the administration of at least one systemic antineoplastic agent in the 8 weeks prior to hospital admission; and an unscheduled hospital admission for inpatient care with eligibility assessed within the first 60 hours of inpatient care. All patients provided written informed consent prior to study inclusion. Patients were excluded if they had undergone surgical treatment within 4 weeks of admission. Unscheduled hospital admission was defined as hospital admission that could not be planned in advance by the health professional due to an acute health event, with the need for urgent medical care that could not be delivered on an ambulatory schedule.

The primary study endpoints were the cumulative incidence of organ failure, defined as the occurrence of any of the following according to the quick Sequential Organ Failure Assessment (qSOFA) criteria: respiratory rate of 22/minute or greater, altered mental status, systolic blood pressure of 100mmHg or less, clinical deterioration that is cause for clinical concern as per the attending medical oncologist or hematologist, and cumulative risk of admission to intensive medical care. The secondary endpoints were the probability of resuming antineoplastic treatment after discharge, survival of cancer patients who developed AOF while undergoing systemic antineoplastic treatment and postdischarge mortality, which was defined as deaths that occurred after hospital discharge. All included patients were treated according to institutional guidelines and local best practices. Data collection for this study was performed after each patient’s hospital discharge. Data were collected with a standardized case report form. This form included patient demographic data, Charlson Comorbidity Index (CCI) variables, cancer-related information (histology, date of diagnosis, disease extent, prior treatment history and last systemic treatment), main diagnosis on inpatient admission criteria, the occurrence of AOF syndrome, the administration of artificial organ replacement therapy (AORT), admission to the ICU, patient health status upon discharge and outcomes. All patients were followed until the end of June 2019.

A sample size of 400 subjects was estimated to allow the computation of the risk of admission to intensive medical care with a precision error of 2% and type 1 error of 5%.^(^14^)^

This study was approved by the hospital administration and Ethics Committee (number CES/IPO: 204/018). All patients consented to participate in this study by providing signed consent forms.

### Statistical analysis

The baseline characteristics of the included subjects at inpatient care admission were described using descriptive statistics as indicated. Two main subgroups were considered, namely, those who had AOF at admission or during the inpatient hospital stay and those who did not have AOF. An exploratory comparison of the baseline characteristics between these subgroups was performed using parametric and nonparametric tests, as appropriate.

The cumulative risk of AOF was calculated as the proportion of patients with AOF at admission or during the inpatient stay out of all patients included in the study. The cumulative risk of admission to the ICU was calculated as the proportion of patients admitted to the ICU out of all patients included in the study.

The outcome of cancer patients who develop AOF while undergoing systemic antineoplastic treatment was evaluated by inpatient hospital mortality, postdischarge mortality and median survival using the Kaplan-Meier method. The outcome data for subjects admitted to the ICU were the mortality rate during the ICU stay and 30 days after discharge from the unit, and the median survival was calculated by Kaplan-Meier method.

Exploratory analyses of the impacts of other baseline characteristics were performed with univariate and multivariate Cox proportional hazard models. An analysis of potential confounders of AOF was performed with a Cox proportional hazard model with the following parameters: age ≥ 60 years old, adjusted CCI ≥ 3, hematologic or nonhematologic malignancy, curative or palliative treatment intent, first or more than 1 line of antineoplastic treatment and admission cause. The proportional hazards assumption was tested using graphical diagnostics based on the scaled Schoenfeld residuals.

No correction for multiple hypothesis testing was established, as this analysis was exploratory and hypothesis generating. All data were analyzed using Statistical Package for Social Sciences (SPSS), version 25.0.

## RESULTS

### Patient and disease characteristics

**Figure 1:**
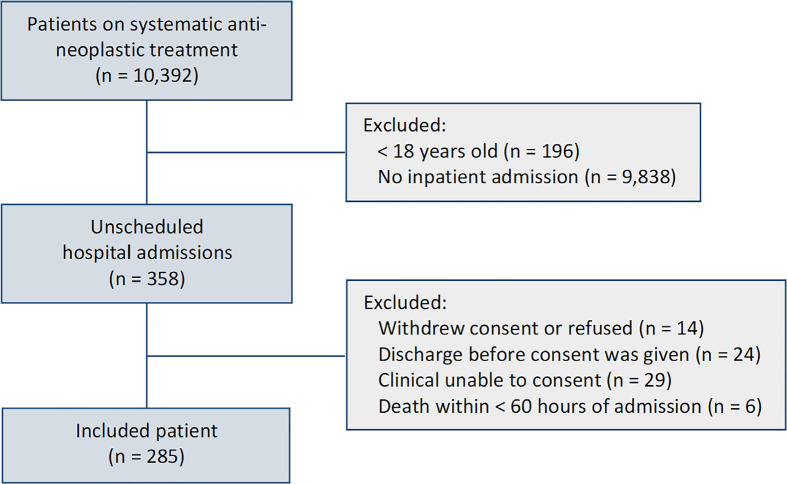
Overview of patients on anti-neoplastic systemic treatment, unscheduled admitted and included in the study.

From August 2018 to February 2019, 10,392 patients were on systemic anti-neoplastic treatment, 358 had unscheduled inpatient care and were eligible for inclusion, and 285 were included (Figure 1). The cohort’s median follow-up duration was 9.5 months (minimum 6 - maximum 12).

The baseline characteristics at the time of acute inpatient admission are described in table 1. The mean age was 60.9 ± 11.8 years, and 50.9% (n = 145) of the subjects were male. The majority (52.3%, n = 149) of patients had significant comorbidities as assessed with the adjusted CCI, and 35.1% (n = 100) were taking 5 or more drugs daily. Hematologic cancer was present in 51 patients (17.9%) and nonhematologic in 234 (82.1%). The most frequent hematologic cancers were nonfollicular lymphoma (37.3%, n = 19), multiple myeloma or malignant plasma cell neoplasms (27.5%, n = 14) and lymphoid leukemia (13.7% n = 7). Regarding the nonhematologic cancers, the most frequent topography of the primary tumor was the digestive tract or glands in 26.5% (n = 62), lungs and respiratory tract in 19.2% (n = 45) and breast in 17.5% (n = 41). Of the 234 nonhematological malignancies, 161 (68.8%) were metastatic at the time of unscheduled hospital admission.

**Table 1:** Baseline characteristics of patients at the time of acute inpatient admission

Characteristic	
Age (years)	60.9 ± 11.8
Male sex	145 (50.9)
Adjusted Charlson comorbidity index	
0	31 (10.9)
1	52 (18.2)
2	53 (18.6)
≥ 3	149 (52.3)
Number of daily drugs in current use	
0	30 (10.5)
1 - 5	155 (54.4)
≥ 5	100 (35.1)
Neoplasia, type	
Nonhematologic	234 (82.1)
Hematologic	51 (17.9)
Primary topography of nonhematologic tumors	
Digestive tract and digestive glands	62 (26.5)
Lungs and respiratory tract	45 (19.2)
Breast	41 (17.5)
Head and neck	14 (6.0)
Gynecologic	14 (6.0)
Others*	58 (24.8)
Type of hematologic tumors	
Nonfollicular lymphoma	19 (37.3)
Multiple myeloma or malignant plasma cell neoplasms	14 (27.5)
Lymphoid leukemia	7 (13.7)
Myeloid leukemia	6 (11.8)
Other†	5 (9.7)
Time since diagnosis (months)	15 [0 - 253]
Current antineoplastic treatment	
Curative intent	76 (26.7)
Time since last treatment (days)	13 [0 - 56]
> 1 previous treatment lines	160 (56.1)
Inpatient admission cause	
Infection	116 (40.7)
Febrile neutropenia	49 (17.2)
Sepsis/septic shock	35 (12.3)
Uncontrolled pain	31(11.2)
Respiratory insufficiency (not infectious)	17 (6.0)
Neurologic dysfunction (not infectious)	15 (5.3)
General status degradation	15 (5.3)
Other causes‡	108 (37.9)

* Malignant neoplasms of mesothelial or soft tissue, malignant neoplasms of male genital organs, malignant neoplasms of urinary tract, malignant neoplasms of skin, malignant neoplasms of thyroid or other endocrine glands, malignant neoplasms of bone and articular cartilage, malignant neoplasms of ill-defined, secondary and unspecified sites, malignant neoplasms of independent (primary) multiple sites; † follicular lymphoma, mature T/NK-cell lymphoma, others and unspecified malignant neoplasms of lymphoid, hematopoietic and related tissue; ‡ disturbances, hemorrhage, hepatic failure, kidney failure, electrolytic disturbances, pancytopenia, cardiac failure or other cardiac causes, cord compression syndrome, mucositis, superior vena cava syndrome, anemia. †,

* , ‡ - frequency less than 5%. Results expressed as mean ± standard deviation, n (%) or median [range].

The median time from the diagnosis of the neoplasia to inpatient admission was 15 months (range 0 - 253), and the median time since the last administration of antineoplastic treatment was 13 days (range 0 - 56). Antineoplastic treatment was prescribed with curative intent in 76 patients (26.7%). The most frequent causes for acute inpatient admission were infections (40.7%, n = 116), followed by uncontrolled pain (11.2%, n = 31) and respiratory insufficiency not attributable to infectious causes (6.0%, n = 17). Of the 116 patients admitted with fever, 56.9% (n=66) had no infection focus identified at admission. The most frequent sites of infection were respiratory (20.7%, n = 24), gastrointestinal (7.8%, n = 9), cutaneous (6.0%, n = 7) and other sites (8.6%, n = 10). At inpatient hospital admission, 62.1% (n = 177) of patients were considered not eligible for artificial organ replacement therapy.

### Risk of acute organ failure and intensive care unit admission

The cumulative risk of AOF at admission for inpatient care was 29.5% (95% confidence interval - 95%CI 26 - 33), and the cumulative risk of AOF (at admission or during inpatient care) was 39.6% (95%CI 35 - 44). For those patients with artificial life support criteria indications at admission, the cumulative risk of AOF was 50.0% (95%CI 41 - 59).

The cumulative risk of the intensive care unit admission of patients with AOF was 15.0% (95%CI 12 - 18). For those patients meeting artificial life support criteria, the cumulative risk of intensive care unit admission of the patients with AOF was 31.5% (95%CI 23 - 40).

The characteristics of and comparisons between patients undergoing systemic antineoplastic treatment who presented with AOF and those who did not are described in table 2. Patients with AOF were 3.3 years older (p = 0.03), with a higher proportion of patients with an adjusted CCI > 3 (62.5% *versus* 45.3%, p = 0.04) and a higher prevalence of hematologic malignancies (25.7% *versus* 12.8%, p = 0.007). Most patients with AOF were in receiving first-line antineoplastic treatment (53.1% *versus* 37.8%, p = 0.015), and there were no differences between the two groups in the intent of treatment (curative or palliative). The most frequent inpatient admission cause of AOF was infection (54.9% *versus* 31.4%, p < 0.001). Of those patients who developed AOF, 34.3% were considered to not benefit from artificial organ replacement therapy.

**Table 2:** Baseline characteristics according to the occurrence of acute organ failure

Characteristic	No AOF n = 172	AOF n = 113	p value
Age (years)	59.6 ± 11.8	62.9 ± 11.6	0.03
Male sex	84 (48.8)	61 (54.0)	0.40
Adjusted CCI			0.04
0	22 (12.8)	9 (8.0)	
1	37 (21.5)	15 (13.4)	
2	35 (20.3)	18 (16.1)	
≥ 3	78 (45.3)	70 (62.5)	
Number of daily drugs in current use			
0	19 (11.0)	11 (9.8)	0.82
1 - 5	95 (55.2)	60 (52.7)	
≥ 5	58 (33.7)	42 (37.5)	
Neoplasia, type			0.007
Nonhematologic	150 (87.2)	84 (74.3)	
Hematologic	22 (12.8)	29 (25.7)	
Primary topography of nonhematologic tumors			
Digestive tract and digestive glands	45 (30.0)	17 (20.2)	0.26
Lungs and respiratory tract	22 (14.7)	23 (27.4)	
Breast	27 (18.0)	14 (16.6)	
Head and neck	9 (6.0)	5 (6.0)	
Gynecologic	9 (6.0)	5 (6.0)	
Others*	38 (25.3)	20 (23.8)	
Previous antineoplastic treatment			0.08
Curative intent	39 (22.7)	37 (32.7)	
Palliative intent	133 (77.3)	76 (67.3)	
Time since last treatment (days)	16 [0 - 56]	17 [0 - 56]	0.88
> 1 previous treatment lines	107 (62.2)	53 (46.9)	0.015
Time since diagnosis (months)	18,5 [0 - 178]	11 [0 - 253]	0.03
Inpatient admission cause			
Infection	54 (31.4)	62 (54.9)	< 0.001
Febrile neutropenia	23 (13.4)	26 (23)	
Sepsis/septic shock	NA	35 (31)	
Uncontrolled pain	26 (15.1)	6 (5.3)	
Respiratory insufficient (not infectious)	5 (2.9)	12 (10.6)	
Neurologic dysfunction (not infectious)	6 (3.5)	9 (8.0)	
General status degradation	12 (7.0)	3 (2.7)	
Other causes†	69 (40.1)	21 (18.6)	
Artificial organ replacement therapy			
Withheld	113 (65.7)	59 (34.3)	0.12

AOF - acute organ failure; CCI - Charlson comorbidity index; NA - not applicable.

* Mesothelial or soft tissue, male genital organs, urinary tract, skin, thyroid or other endocrine glands, bone and articular cartilage, ill-defined, secondary and unspecified sites, neoplasms of independent (primary) multiple sites; † gastrointestinal disturbances, hemorrhage, hepatic failure, kidney failure, electrolytic disturbances, pancytopenia, cardiac failure or other cardiac causes, cord compression syndrome, mucositis, superior vena cava syndrome, anemia.

* and † frequency less than 5%. Results expressed as mean ± standard deviation, n (%) or median [range].

### Characteristics of patients admitted to the intensive care unit

Of the 17 patients admitted to the ICU, 23.5% (n = 4) had hematologic cancers, 17.6% (n = 3) had digestive tract cancer, 17.6% (n = 3) had breast cancer, 17.6% (n = 3) had male genital cancer, 11.8% (n = 2) had lung cancer, 5.9% (n = 1) had hypopharyngeal cancer, and 5.9% (n = 1) had small intestine neuroendocrine cancer. Antineoplastic systemic treatment was administered with curative intent to 8 patients (47.1%). Acute organ failure was present at hospital admission in 13 patients (76.5%). The main diagnosis on ICU admission was infection (58.9%; n = 10), febrile neutropenia (29.4%; n = 5), sepsis or septic shock (41.2%; n = 7), and noninfectious respiratory insufficiency (11.8%; n = 2); there was one case each of neurologic dysfunction, cardiac insufficiency, acute renal failure, carcinoid syndrome and perforated hollow viscus.

### Patient outcomes

**Figure 2:**
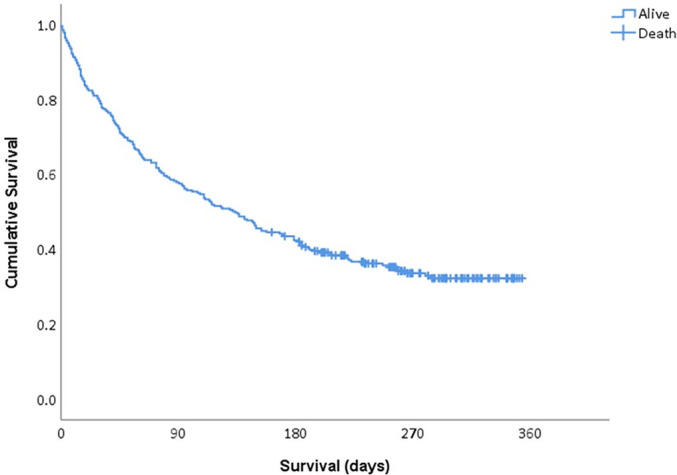
Median survival of patients with an unscheduled hospital admission.

Overall, in-hospital mortality was 17.5%, and among those patients admitted to the ICU, in-hospital mortality was 58.8%. Of those patients discharged home, 63.8% resumed antineoplastic treatment. Of the patients who required ICU care, 57.1% resumed antineoplastic treatment. In univariate analysis, the probability of resuming systemic therapy was higher among those patients being treated with curative intent for their cancer, those who had improved health status at the time of discharge and those with hematologic cancers (Table 3). The median survival duration was 134 days (95%CI 106 - 162), with an overall mortality rate of 65.6% (n = 187) (Figure 2). The median survival duration for the ICU-admitted patients was 73 days (95%CI 0 - 163).

**Figure 3:**
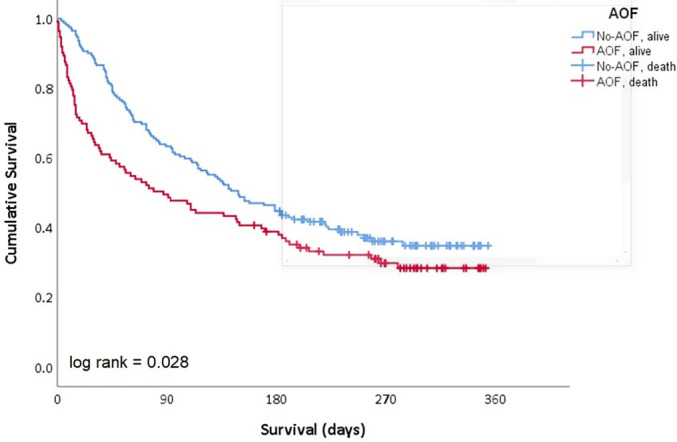
Median survival of patients according to the occurrence of acute organ failure.

Patients who developed AOF had a median survival duration of 87 days (95%CI 41 - 133), which was significantly lower than that of patients without AOF (median 149 days; 95%CI 110 - 188; p = 0.028) (Figure 3). Acute organ failure was associated with both an increased risk of in-hospital mortality, hazard ratio (HR) 3.4; 95%CI 1.8 - 6.5; p < 0.0001, and increased postdischarge mortality, HR 1.6 (95%CI 1.2 - 2.2, p = 0.002), after adjustment for the following covariates: age ≥ 60 years old, adjusted CCI ≥ 3, hematologic or nonhematologic malignancy, curative or palliative treatment intent, first or more than 1 line of antineoplastic treatment and admission cause. The proportional hazards assumption was met for all the covariates used in the Cox model.

**Table 3:** Baseline characteristics between patients who resumed systemic treatment and those who did not

Characteristic	No systemic treatment resumed n = 85	Resumed systemic treatment n = 150	p value
Age (years)	61.7 ± 10.5	59.8 ± 11.7	0.20
Male Sex	46 (40.0)	69 (60.0)	0.28
Adjusted CCI			0.49
0	7 (28.0)	18 (72.0)	
1	19 (43.2)	25 (56.8)	
2	15 (30.6)	34 (69.4)	
≥ 3	44 (37.6)	73 (62.4)	
Number of daily drugs in current use			0.75
0	11 (42.3)	15 (57.7)	
1 - 5	47 (36.4)	82 (63.6)	
≥ 5	27 (34.2)	52 (65.8)	
Neoplasia, type			0.03
Nonhematologic	76 (39.4)	117 (60.6)	
Primary topography of nonhematologic tumors			0.18
Digestive tract and digestive glands	30 (54.5)	25 (45.5)	
Lungs and respiratory tract	10 (31.3)	22 (68.8)	
Breast	12 (32.4)	25 (67.6)	
Head and neck	6 (50.0)	6 (50.0)	
Gynecologic	2 (18.2)	9 (81.8)	
Others*	16 (34.8)	30 (65.2)	
Previous antineoplastic treatment			
Curative intent	15 (21.7)	54 (78.3)	0.004
Palliative intent	70 (42.2)	96 (57.8)	
Time since last treatment (days)	13.0 [0 - 56]	12.0 [0 - 56]	0.34
> 1 previous treatment lines	53 (40.8)	77 (59.2)	0.13
Time since diagnosis (months)	18.0 [0 - 150]	14.5 [0 - 215]	0.47
Inpatient admission cause			0.07
Infection	26 (26.0)	74 (74.0)	
Uncontrolled pain	11 (40.7)	16 (59.3)	
Respiratory insufficient (not infectious)	7 (53.8)	6 (46.2)	
Neurologic dysfunction (not infectious)	2 (25.0)	6 (75.0)	
General status degradation	4 (50.0)	4 (50.0)	
Other causes†	35 (44.3)	44 (55.7)	
AOF	29 (37.7)	48 (62.3)	0.77
Discharge patient health status			0.04
Improved	64 (32.8)	131 (67.2)	
Stable	18 (50.0)	18 (50.0)	
Worsen	3 (75.0)	1 (25.0)	

CCI - Charlson comorbidity index; AOF - acute organ failure.

* Malignant neoplasms of mesothelial or soft tissue, malignant neoplasms of male genital organs, malignant neoplasms of urinary tract, malignant neoplasms of skin, malignant neoplasms of thyroid or other endocrine glands, malignant neoplasms of bone and articular cartilage, malignant neoplasms of ill-defined, secondary and unspecified sites, malignant neoplasms of independent (primary) multiple sites. † gastrointestinal disturbances, hemorrhage, hepatic failure, kidney failure, electrolytic disturbances, pancytopenia, cardiac failure or other cardiac causes, cord compression syndrome, mucositis, superior vena cava syndrome, anemia. Results expressed as mean ± standard deviation, n (%) or median [range].

AOF - acute organ failure.

## DISCUSSION

We conducted a prospective cohort study that included cancer patients treated with systemic anti-neoplastic therapies in the largest Portuguese comprehensive cancer center during a six-month period to assess the cumulative risk of AOF and the cumulative incidence of ICU admission while on treatment. We estimate the risk of AOF on hospital admission in patients undergoing systemic anticancer treatment to be 29.5% and the risk of ICU admission to be 15%. To our knowledge, this is the first published study addressing the risk of developing AOF in cancer patients while receiving ambulatory anti-neoplastic systemic treatment. The determination of the incidence of AOF in cancer patients is of particular interest because it may impact short-term survival and lead to higher medical resource use due to the referral of patients for ICU care.^(^7^,^8^)^

Most studies addressing acutely ill cancer patients are focused on patients admitted for intensive medical care and their short-term outcomes (e.g., in-hospital mortality, 28-day mortality).^(^7^,^8^,^15^)^ These studies are commonly retrospective and have heterogeneous patient samples, including different case mixes of medical and surgical patients, hematologic and solid cancer patients and bone marrow transplant recipients. Our sample included cancer patients receiving systemic antineoplastic treatment with an unscheduled hospital admission while on-treatment, with the main purpose being to evaluate the incidence of AOF in these patients. We cannot rule out selection bias resulting in the possible underestimation of these risks, as the accrual of patients was based on single-center recruitment, and some patients whose treatment had been prescribed at our center may have been admitted for acute care treatment at other hospitals or died at home due to AOF. Moreover, some patients who were clinically unable to consent or were discharged or died before being able to sign the consent form for study participation were not included; however, the impacts of these factors on our estimates are uncertain.

In our study, patients with older age, adjusted CCI ≥ 3 and hematological diseases were more likely to have AOF when admitted for unscheduled inpatient care after systemic cancer therapy. This was more frequent during first-line systemic treatment and in patients with a shorter time interval from the diagnosis of cancer, which is probably related to the aggressiveness of first treatment lines in patients whose baseline biological reserves are yet unknown.^(^16^)^ Infection was the primary reason for unscheduled hospitalization, and of these patients, 17.2% presented with febrile neutropenia and 12.3% with sepsis or septic shock, thus contributing to the high prevalence of AOF.

The choice of the qSOFA score as the outcome measure for defining AOF was based on its ease of applicability and its status as a validated measure associated with in-hospital mortality in a non-ICU setting in patients with confirmed or suspected infection.^(^17^-^19^)^ Additionally, in the noninfectious context, it has been prospectively studied for the assessment of acute organ failure with 2-day and 30-day mortality prognostic accuracies of 79.9% and 76.2%, respectively.^(^20^)^ The qSOFA has also been prospectively compared against the systemic inflammatory response syndrome (SIRS) score for the prediction of ICU and hospital mortality in critically ill cancer patients, with better prognostic accuracy than SIRS for both parameters.^(^20^)^ Therefore, we believe that the outcome measurement and adjudication method did not bias the estimated risk of AOF in these patients.

When considering the entire hospitalization period, the cumulative risk of AOF increased to 39.6%. The reported risk of sepsis of 12.3% is comparable with prior estimates that ranged from 4.9% and 46% in ICU-admitted cancer patients.^(^21^,^22^)^ When considering AORT, 1 in 3 patients who developed AOF were admitted for ICU care, 75% of whom were on the first day of their hospital stays, and half of these patients were undergoing treatment with curative intent. Infection was the primary diagnosis at ICU admission, and ICU-admitted patients had an in-hospital mortality rate in excess of 50%. These estimates are consistent with previous findings, particularly when considering the studies that included hematological cancer patients. For instance, one study found the incidence of ICU mortality in solid cancer patients to be 31% and the incidence of in-hospital mortality in admitted ICU patients to be 38%, with outcomes depending on cancer topography, type of admission (planned or emergency) and specialty.^(^23^)^ Another study found the incidences of ICU and in-hospital mortality rates for hematological cancer patients to be 24.8% and 45.3%, respectively.^(^24^)^

Overall, in-hospital mortality was 17.5%, and the survival of these patients was poor, with a median survival duration of 4.5 months among all patients and 2.5 months among those admitted for ICU care. The occurrence of AOF was associated with a 3-fold increase in mortality and a 2-fold increase in mortality after hospital discharge even after adjustment for age and comorbidity. This higher mortality for patients who developed AOF is probably not directly related to the acute event but rather inherent to the patient’s condition or subsequent treatment decisions. Patients with a previous episode of AOF while receiving treatment may be at increased risk of subsequent hospitalizations, with a higher likelihood of a serious adverse event and death. On the other hand, the occurrence of AOF may lead to changes in the patient’s therapeutic plan, with dose reductions and changes in or the suspension of treatment, which may be associated with decreased survival. Despite the worse prognosis of patients who developed AOF while receiving systemic medical treatment, patients undergoing potentially curative therapy and those with advanced cancer with predictable long-term survival may benefit from ICU admission.^(^5^)^ For patients with advanced cancer, an ICU trial can be valuable, as it can potentially prolong survival with good quality of life.^(^25^)^ Although ICU admission recommendations for critically ill cancer patients have been proposed by an international expert consensus, there are no established criteria for the ICU admission of oncologic patients.^(^4^)^ In future, we intend to design and study the applicability of a protocol with pre-established admission criteria for critically ill cancer patients in the ICU.

## CONCLUSION

In this single-center prospective cohort study, cancer patients who required unscheduled inpatient medical care had a cumulative risk of acute organ failure of 39.6% and a 15% risk of the need for intensive medical care treatment. Acute organ failure was associated with increased mortality both during the hospital stay and after discharge.

### Availability of data and materials

The datasets generated and analyzed during the current study are not publicly available due to Union European General Data Protection Regulations but are available from the corresponding author upon reasonable request after approval by the institutional ethics committee and by the local government responsible for assessing the impact of data protection.
